# Effect of Acceptance and Commitment Therapy on Motivation and Adherence to Immunosuppressive Medication in Liver Transplant Recipients: A Randomized Controlled Trial

**DOI:** 10.1002/brb3.70882

**Published:** 2025-09-29

**Authors:** Serdar Saritas, Serafettin Okutan, Hasan Saritas, Semra Bulbuloglu

**Affiliations:** ^1^ Department of Medical Biology, Faculty of Medicine Malatya Turgut Ozal University Malatya Turkey; ^2^ Division of Surgical Nursing, Nursing Department, Faculty of Health Sciences Bitlis Eren University Bitlis Turkey; ^3^ Division of Surgical Nursing, Nursing Department, Faculty of Health Sciences Siirt University Siirt Turkey; ^4^ Department of Midwifery, Faculty of Health Sciences Istanbul Aydin University Istanbul Turkey

**Keywords:** acceptance and commitment therapy, immunosuppressive, liver transplant, medication, motivation, transplantation, treatment adherence

## Abstract

**Background:**

After liver transplantation, recipients continue to receive immunosuppressive drug regimens at varying doses for years to ensure graft function and survival. Although liver transplant recipients are aware of the importance of immunosuppressive drug regimens, treatment compliance may decrease over time due to low motivation, putting recipients at risk of organ rejection. Acceptance and commitment therapy (ACT) appears to be an ideal solution for increasing motivation and, indirectly, treatment compliance.

**Objective:**

The aim of this study was to investigate the effect of ACT on adherence to immunosuppressive medication and motivation in liver transplant recipients.

**Methods:**

This study was conducted at an organ transplant hospital with the participation of recipients who had completed at least 6 months post‐liver transplant. The sample included *n* = 140 liver transplant recipients (*n* = 70 experimental, *n* = 70 control). The study was randomized and controlled. The control group received no intervention, while the experimental group underwent an eight‐session ACT program administered by a psychologist. Data collection utilized a personal characteristics form, the Adherence to Immunosuppressive Drug Regimen Scale (AIDRS), the Mindfulness Scale (MS), and the Adult Motivation Scale (AMS). Data analysis was performed using descriptive statistical methods and tests of significance for differences between variables.

**Findings:**

According to the homogeneity tests of this study, there was no statistically significant difference between the control and experimental groups in terms of individual characteristics (age, time since transplantation, immunosuppressive drugs, comorbidities, etc.). In the experimental group, the level of adherence to the immunosuppressive drug regimen after ACT was very close to the maximum AIDRS score. In the control group, the level of compliance with the immunosuppressive drug regimen was close to the mean value of the AIDRS. The differences mentioned in both groups were statistically significant (*p* < 0.05). In addition, the scores obtained from the MS and YMS were higher than those in the pretest. When the experimental and control groups were compared, the experimental group's final test YMS score was almost twice that of the control group, and this difference was statistically significant (*p* < 0.05).

**Conclusion:**

The importance of full compliance with the immunosuppressive drug regimen in improving graft function after liver transplantation is well known. The results of this study showed that liver transplant recipients who underwent ACT demonstrated higher adherence to the immunosuppressive drug regimen due to increased motivation and conscious awareness. We recommend the use of ACT to increase adherence to the immunosuppressive medication due to its effectiveness.

## Graphical abstract

A total of 140 liver transplant recipients were included in the experimental and control groups of this study. Recipients in the experimental group received eight sessions of acceptance and commitment therapy (ACT). Adherence to immunosuppressive medication and motivation increased in the ACT‐treated experimental group, while medication adherence decreased in the control group, which received no intervention (*p* < 0.05). ACT is an effective method for improving graft function after liver transplantation and increasing adherence to immunosuppressive medication and motivation.

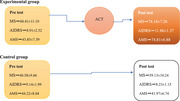

## 1 Introduction

Thanks to modern surgical techniques, imaging methods, and effective immunosuppressive drug regimens, morbidity has decreased and survival rates have increased after liver transplantation (Craig and Heller 2021). Transplant surgery is a therapeutic and curative treatment option for end‐stage chronic liver disease (Sousa Da Silva et al. 2022). Currently, the 1‐year survival rate after liver transplantation is over 90% (Kwong et al. 2022). A functional graft in the recipient's body may offer the potential for a lower dose and variety of immunosuppression regimens by regulating the recipient's immune system to promote tolerance. The selection and regulation of immunosuppressive agents are personalized to reduce toxicity and optimally control alloreactivity (Montano‐Loza et al. 2023). Low, less varied, and individualized immunosuppressive drug regimens are an important convenience provided by physicians to liver transplant recipients. A previous study noted that immunosuppressive drugs were prescribed by physicians, yet graft rejection was commonly observed (Gordon et al. 2007), with failed graft outcomes often attributed to recipients' non‐compliance with years‐long treatments and the complexity of drug dosages (Zhu et al. 2017). Several studies have found that organ recipients do not adhere to immunosuppressive treatment at the desired level (Bulbuloglu and Demir 2021; Demir and Bulbuloglu 2021; Goldfarb‐Rumyantzev et al. 2010). Failure to adhere to the immunosuppressive treatment regimen in liver transplant recipients may increase the risk of mortality.

In liver transplant recipients, a longer life span is inevitable with high self‐care. During this process, it is very important for the patient to have high energy and motivation and to want to live more than anyone else. However, increased needs after liver transplantation, the economic burden caused by health problems, lack of social support, and physical limitations can lead to exhaustion in recipients (Bulbuloglu et al. 2023; Harmancı et al. 2023; Harmanci and Bulbuloglu 2023; Müller et al. 2025). Therefore, there is a need for strategies that will connect liver transplant recipients to life and increase their motivation. In studies involving individuals with a need for regular medication use, anxiety, depression, lack of self‐control, cognitive, and behavioral issues were managed through psychoeducational and mindfulness‐based interventions, thereby improving patients' treatment adherence (Nardi et al. 2021; Rusch et al. 2019; Scott‐Sheldon et al. 2019; Torbati, Abbaspour, et al. 2022; Torbati, Zandi, et al. 2022; Q. Zhang et al. 2019). 10% of hospital readmissions are due to issues arising from non‐adherence to medication (Osterberg and Blaschke 2005), and in the United States, preventable hospital readmissions are associated with an additional economic burden of up to $289 billion annually (Cutler et al. 2018). In Turkey, the total cost of comorbid conditions to the Turkish economy was recorded as 17.87 billion dollars in 2016 (Medimagazin 2018). Common therapies rooted in cognitive awareness include mindfulness‐based stress reduction, cognitive therapy, acceptance and commitment therapy, and dialectical behavior therapy (Crane et al. 2017). In this study, we aimed to investigate the effect of acceptance and commitment therapy on adherence to immunosuppressive medication and motivation in liver transplant recipients.

## 2 Materials and Methods

### 2.1 Study Design and Sample

**FIGURE 1:**
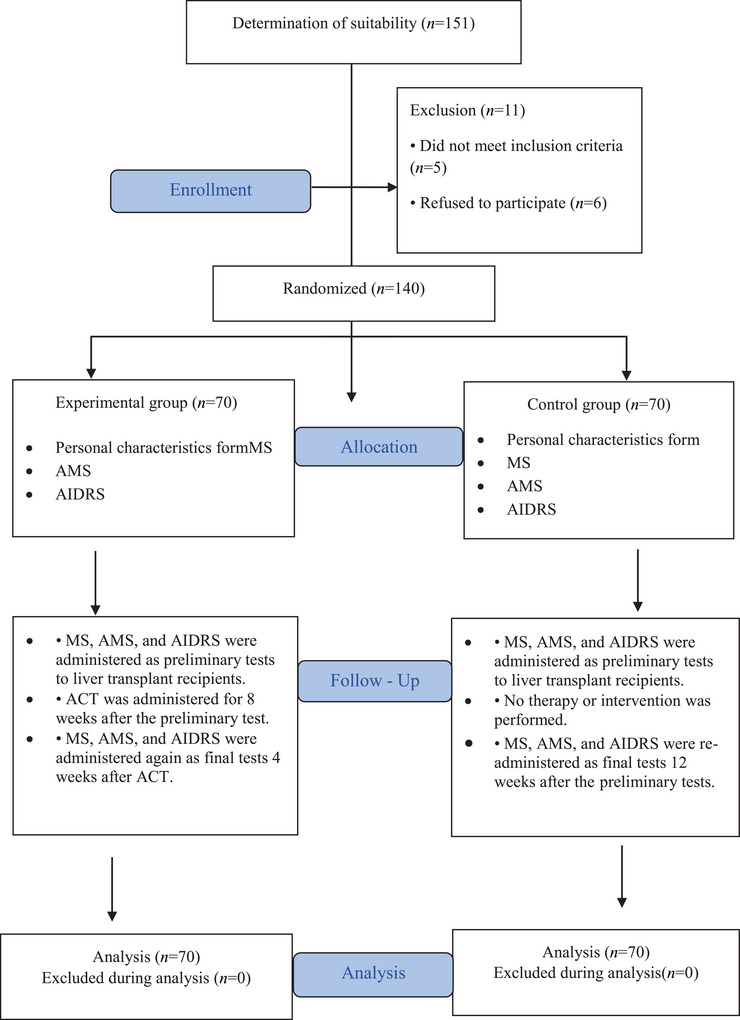
Research flow chart.

In this study, we investigated the effectiveness of ACT in improving compliance with immunosuppressive drug regimens and motivation levels in liver transplant recipients. We followed the steps of the Consolidated Standards of Reporting Trials (CONSORT) statement in this study (Moher et al. 2024). The research flow chart is shown in Figure 1. This study was a randomized controlled trial. The data collection period was February 15, 2025–May 30, 2025. Data collection was conducted by researchers at the organ transplantation institute of an education and research hospital in eastern Turkey. A psychologist was hired to administer the ACT. The sample consisted of liver transplant recipients who met the inclusion and exclusion criteria. Approximately 250–300 liver transplants are performed annually at the organ transplantation hospital where the study was conducted. To calculate the sample size, the G Power 3.1.9.7 software program was used. According to this program, a minimum of 116 participants were required with an effect size of 0.8, a margin of error of 0.05, a confidence interval of 0.95, and a power of 95% to represent the population. A total of 140 recipients were included in the experimental and control groups of this study. Eleven patients who did not meet the inclusion criteria and/or were unwilling to participate in the study were excluded from the sample (Figure 1). The inclusion and exclusion criteria for the sample are shown below.

### 2.2 Inclusion and Exclusion Criteria

The inclusion criteria for this study were (i) liver transplant recipients were offered participation in the study during routine outpatient follow‐up and accepted the offer (liver transplant recipients agreed to come to special consultation rooms at intervals requested by the researchers), (ii) having been on an immunosuppressive drug regimen for at least 6 months, (iii) being 18 years of age or older, with no communication issues, (iv) recipients who had undergone primary liver transplantation, and (v) not being a participant in another study where a similar initiative was being conducted at the same time. The opposite of these criteria was accepted as the exclusion criterion.

### 2.3 Data Collection Tools

The data collection tools administered to liver transplant recipients included a personal characteristics form, the Immunosuppressive Drug Regimen Adherence Scale, the Mindfulness Awareness Scale, and the Adult Motivation Scale (AMS). Information regarding the data collection tools is provided below.

### 2.4 Personal Information Form

The personal information form was developed by researchers based on the literature (Bulbuloglu et al. 2023; Demir and Bulbuloglu 2021; Goldfarb‐Rumyantzev et al. 2010; Harmanci and Bulbuloglu 2023). It is a data collection tool that inquires about the socio‐demographic characteristics of liver transplant recipients (age, gender, marital status, education level, etc.), immunosuppressive drug use, and possible problems.

### 2.5 Adherence to Immunosuppressive Drug Regimen Scale

AIDRS was first used by Morisky and colleagues in 1986 to assess adherence to antihypertensive medications. Chisholm and colleagues adapted the same scale for organ transplant patients in 2004 (Chisholm 2002; Chisholm et al. 2005). The AIDRS consists of four questions, each of which assesses how many times and why immunosuppressive drugs that prevent organ rejection were missed in the past 3 months. During scoring, organ transplant recipients receive 3 points if they have not missed any immunosuppressive medication treatment in the past 3 months, 2 points if they have missed 1%–20%, 1 point if they have missed 21%–50%, and 0 points if they have missed > 50%. The AIDRS score range is 0–12, with higher scores indicating better medication adherence. According to the validity and reliability analyses of the original scale, the results are reported to be optimal (Madran et al. 2016). The Turkish validity and reliability study of AIDRS was conducted by Madran et al. (2016), and according to this study, AIDRS was found to be valid and reliable (Madran et al. 2016).

### 2.6 Mindfulness Scale (MS)

The MS was developed by Brown and Ryan (2003) and is used to measure mindfulness levels. The MS consists of 15 items, is a 5‐point Likert scale, and is evaluated based on a single total score; it has no subscales. The MS score range is between 15 and 75. Higher scores indicate higher levels of mindfulness. The internal consistency coefficient of the original scale was found to be 0.82, and the test‐retest reliability was 0.81 (Brown and Ryan 2003). The MS was adapted into Turkish by Özyeşil et al. (2011). The internal consistency coefficient of the Turkish version is 0.80, and the test‐retest reliability is 0.86 (Özyeşil et al. 2011). In our study, we found that the Cronbach's alpha coefficient was 0.81.

### 2.7 Adult Motivation Scale

The AMS was developed by Tulunay‐Ateş and İhtiyaroğlu in 2019 (Ateş and İhtiyaroğlu 2019). The scale consists of two subscales: intrinsic and extrinsic motivation. The AMS has a score range of 21–105 and consists of 21 questions. The internal motivation subscale has a score range of 13–65 and consists of 13 questions. The score range for the external motivation sub‐dimension is between 8 and 40, and there are eight questions. There are no reverse questions in the MS, and it is a 5‐point Likert‐type scale. It is stated that the reliability value of the internal motivation sub‐dimension in the MS is 0.92, while that of external motivation is 0.82. The total reliability value of the MS is 0.94, and the two subscales together explain 47.95% of the total variance. In this study, the internal consistency Cronbach's alpha value of the MS was determined to be 0.91 for the internal motivation subscale, 0.80 for external motivation, and 0.86 for the total scale.

### 2.8 Randomization

Each liver transplant recipient was assigned to the experimental and control groups using a simple random sampling method, and a web‐based Random Assignment Software (RAS) was used for this purpose (Arslan et al. 2019). Before liver transplant recipients began home follow‐up, immunosuppressive medications were prescribed by a clinician, and nurses provided education on the immunosuppressive medication regimen. In addition, all liver transplant recipients were informed about the purpose of immunosuppressive medications. In our study, all liver transplant recipients in the experimental and control groups had been using immunosuppressive drugs for at least 6 months. The responsibility for adhering to the immunosuppressive drug regimen lay with the patients themselves after they were discharged home, and it was monitored by physicians and nurses at each outpatient clinic visit. The sample consisted of *n* = 140 individuals, divided into two groups: the experimental and control groups, with 70 liver transplant recipients in each group. The information for the experimental and control groups is shown below.

### 2.9 Experimental Group

Prior to acceptance and commitment therapy, the recipients in the experimental group were administered a personal characteristics form, as well as the MS, AMS, and AIDRS as pre‐tests. The 70 liver transplant recipients in the experimental group were divided into seven groups of 10 people each. ACT was administered to each group on different days. Table 1 includes a summary of the ACT Protocol. During the therapy, the following themes were addressed in sequence: “Introduction and Presentation of the ACT‐Based Program,” “Values,” “Creative Hopelessness, Control is a Problem,” “Cognitive Defusion,” “Understanding, Connecting, and Accepting,” “Contextual Self,” “Behaviors Aligned with Values,” and “Closing.” ACT was administered once a week for 8 weeks, with each session lasting three hours, and feedback was collected from each participant at the end of each session. After the 8‐week ACT program was completed, a 4‐week break was taken, and at the end of these 4 weeks, the MS, AMS, and AIDRS were administered as final tests.

**TABLE 1:** Summary of the acceptance and commitment therapy protocol.

Module	Content
1	Introduction and Presentation of the ACT‐Based Program: The purpose of the study was explained, the ACT‐based program was introduced, and informed consent was obtained. ACT sessions were explained, and a therapist agreement was reached.
2	Values: Discussing past experiences and observations, principles of being a benchmark, developing creative despair, practicing understanding and accepting feelings and thoughts, doing healing exercises, measuring willingness to change, summarizing, and homework.
3	Creative Hopelessness, Control is a Problem: Recognition of overcontrol as a problem and presentation of willingness as a possible response, undertaking purposeful actions, attracting valued aspects, giving up anxiety control and accepting value‐based behavior, discussion of risks and difficulties in accepting illness and treatment, planning of exercises to be done in the next session, and homework.
4	Cognitive Defusion: Cognitive defusion techniques are used in this phase. Problematic language chains are broken down, the ties between the self and thoughts and emotions are weakened, awareness‐based acceptance of anxiety is learned, and the distinction between internal and external control is addressed, as well as the functioning of problematic metaphors and language chains.
5	Understanding, Connecting, and Accepting: Accepting oneself as a context and observing, stretching the conceptualized self‐construct and strengthening the observing self, drawing the context‐accepted self‐construct into oneself, and learning from anxious and fearful thoughts and feelings.
6	Contextual Self: Use of mindfulness techniques, modeling the “mind” as an independent entity, accepting that gaining experience is a process, understanding the importance of emotions and experiences in enriching life, identifying and focusing on life values, summarizing, and planning exercises and homework for the next session.
7	Behaviors Aligned with Values: Introducing and explaining the concept of value, understanding the disadvantages of focusing on results, revealing practical values in life, identifying the differences between values and goals and common mistakes, and identifying internal and external obstacles.
8	Closing: Understanding the nature of ACT involves knowing values‐based action patterns, developing values‐focused behavior plans, and making efforts to create commitment to them.

### 2.10 Control Group

The liver transplant recipients in the control group were administered the MS, AMS, and AIDRS as pre‐tests along with a personal information form. Each liver transplant recipient with questions was given the opportunity to discuss them comfortably with the researchers. No other interventions were made with the control group, and no follow‐up visits were conducted over the 12‐week period. At the end of 12 weeks, liver transplant recipients in the control group were invited to the outpatient clinic, and the MS, AMS, and AIDRS were administered as the final test during their outpatient visits.

### 2.11 Data Analysis

The data from this study were analyzed using the Statistical Package for the Social Sciences (SPSS) 27.0 IBM (Armonk, NY) program. A 95% confidence interval and a statistical significance level of *p* < 0.05 were used in the data evaluation. Descriptive statistical tests were used to categorize individual characteristics and calculate averages. The normal distribution of the data were determined using the Kolmogorov–Smirnov test, and it was found that the data did not follow a normal distribution. The homogeneity of individual characteristics of the experimental and control groups was checked using the chi‐square test and Mann–Whitney *U* test. The Wilcoxon test and Mann–Whitney *U* test were used to compare scale scores within and between groups. The Cronbach alpha Coefficient was taken into account in determining the validity and reliability levels of the scales.

### 2.12 Ethical Considerations

Prior to the start of this study, Institutional Review Board approval was obtained from the Turgut Özal Medical Center Liver Transplant Institute. Subsequently, Ethical Committee approval was obtained from the Bitlis Eren University Ethics Committee (Date: February 13, 2025, Decision No: 2025/1‐2). In the research protocol, informed written consent was obtained from each liver transplant recipient, taking into account compliance with the Helsinki Declaration. Additionally, the study protocol was recorded on the Clinical Trials Platform with the registration number NCT06879106.

## 3 Findings

Table 2 shows the results of descriptive tests regarding the personal characteristics of liver transplant recipients and homogeneity tests performed between the control and experimental groups. Homogeneity tests conducted between the control and experimental groups revealed no statistically significant differences in terms of age, time elapsed since transplantation, gender, education and marital status, economic status, having children, immunosuppressive drugs used, and comorbidities (*p* > 0.05). Based on these results, the individual characteristics of the control and experimental groups were similar, and there was a homogeneous distribution.

**TABLE 2:** Individual characteristics and homogeneity tests of liver transplant recipients (*N* = 140).

Individual characteristics	Control group (*n* = 70)		Experimental group (*n* = 70)		Homogeneity test and Sig.
Means	($\bar{x}$ +Sd.)	Min‐Max	($\bar{x}$ +Sd.)	Min‐Max	
**Age**	40.22 ± 6.86	28‐56	41.77 ± 6.72	28‐56	*U* = 2,134, *p* = 0.187
	***n***	**%**	***n***	**%**	
**Days after transplant**					
6 months	7	10	4	5.7	χ^2^ = 8,757, *p* = 0.058
Between 6 months and 1 year	45	64.3	45	64.3	
Between 1 and 2 years	18	25.7	21	30	
**Gender**					
Female	17	24.3	11	15.7	χ^2^ = 2,065, *p* = 0.082
Male	53	75.7	59	84.3	
**Educational status**					
Elementary school	18	25.7	17	24.3	χ^2^ = 5,243, *p* = 0.073
High school	16	22.9	32	45.7	
University	36	51.4	21	30	
**Marital status**					
Single	22	31.4	16	22.9	χ^2^ = 2,240, *p* = 0.256
Married	48	68.6	54	77.1	
**Economic status**					
Low	20	28.6	14	20	χ^2^ = 6,342 *p* = 0.067
Medium	40	57.1	44	62.9	
High	10	14.3	12	17.1	
**Being a parent**					
Yes	53	75.7	54	77.1	χ^2^ = 2,415, *p* = 0.843
No	17	24.3	16	22.9	
**Immunosuppressive medication use**					
Corticosteroid	66	94.3	62	88.6	*U* = 2,192, *p* = 0.223
Azathioprine	24	34.3	21	30	*U* = 1,836, *p* = 0.191
Cyclosporine	25	35.7	27	38.6	*U* = 2,340, *p* = 0.597
Tacrolimus	46	65.7	48	68.6	*U* = 2,310, *p* = 0.500
Mycophenolate mofetil	38	54.3	36	51.4	*U* = 2,450, *p* = 0.914
Sirolimus	18	25.7	19	27.1	*U* = 2,412, *p* = 0.861
**Comorbidity**					
Chronic kidney disease	5	7.1	4	5.7	*U* = 2,345, *p* = 0.613
Hypertension	25	35.7	22	31.4	*U* = 2,188, *p* = 0.221
Asthma‐COPD	4	5.7	5	7.1	*U* = 2,188, *p* = 0.866
Diyabetes mellitus	7	10	7	10	*U* = 2,415, *p* = 0.550
Other (neurological, intestinal, and cardiovascular problems)	24	34.3	22	31.4	*U* = 2,345, *p* = 0.529

*Note: χ* ^2^ = chi‐square test, *U* = Mann–Whitney *U* test.

Table 3 shows a comparison of the experimental and control groups' compliance with immunosuppressive medications, conscious awareness, and motivation levels. There was no statistically significant difference between the experimental and control groups' AIDRS, MS, and AMS scores before ACT. After ACT, the AIDRS, MS, and AMS scores increased in the experimental group, and the intra‐group differences were statistically significant (*p* < 0.01). The mean scores for the internal and external motivation subscales of AMS increased to a statistically significant level after ACT (*p* < 0.01). The control group's final test AIDRS score was lower than the pre‐test score, and this difference was statistically significant (*p* = 0.001). The control group's final test MS score was lower than the pre‐test score, but the minimal score difference between them was not statistically significant (*p* = 0.558). The control group's internal and external motivation scores, as well as their AMS total scores, were lower in the final test compared to the pre‐test, but these differences were not statistically significant (*p* > 0.05).

**TABLE 3:** Comparison of immunosuppressive medication compliance, mindfulness, and motivation levels in the experimental and control groups.

Scales	Measurement time	Experimental group	Control group	Comparison of two groups
		$\bar{x}$ +Sd. (min, max)	$\bar{x}$+Sd. (min, max)	Test and Sig.
**AIDRS**	Pre test	8.91 ± 2.32 (5, 11)	9.14 ± 1.99 (6, 11)	*U* = 1,879, *p* = 0.059
	Final test	11.86 ± 1.37 (11, 12)	8.23 ± 1.15 (5, 12)	*U* = 2,041, ***p* = 0.005** ^**^
	**Comparison of repeated measurements**	*Z* = 1,414, *p* = 0.003^**^	*Z* = 3,039, *p* = 0.001^**^	
**MS**	Pre test	60.41 ± 11.10 (40, 64)	60.58 ± 9.66 (43, 66)	*U* = 14,701, *p* = 0.638
	Final test	74.18 ± 7.26 (68, 79)	59.13 ± 10.24 (36, 64)	*Z* = 10,022, *p* = 0.001^**^
	**Comparison of repeated measurements**	*Z* = 2,364, *p* = 0.001^**^	*Z* = 1,071, *p* = 0.558	
**AMS**	**AMS total**			
	Pre test	43.85 ± 7.39	44.22 ± 8.04	*U* = 0.137, *p* = 0.712
	Final test	74.81 ± 6.88	41.97 ± 6.74	*U* = 0.257, *p* = 0.011^*^
	**Comparison of repeated measurements**	*Z* = 0.691, *p* = 0.001^**^	*Z* = 14,712, *p* = 0.077	
	**Internal motivation**			
	Pre test	24.41 ± 6.62	24.10 ± 5.32	*U* = 1,255, *p* = 0.314
	Final test	46.31 ± 3.47	23.18 ± 7.17	*U* = 2,512, *p* = 0.003^**^
	**Comparison of repeated measurements**	*Z* = 0.721, *p* = 0.001^*^	*Z* = 2,609, *p* = 0.091	
	**External motivation**			
	Pre test	19.24 ± 5.88	19.72 ± 4.90	*U* = 3,098, *p* = 0.585
	Final test	28.40 ± 6.70	18.85 ± 6.01	*U* = 1,944, *p* = 0.001^**^
	**Comparison of repeated measurements**	*Z* = 0.756, *p* = 0.009^*^	*Z* = 1,534., *p* = 0.656	

^Note: Z^ = Wilcoxon test, *U* = Mann–Whitney *U* test.

^**^ *p* < 0.01 and ^*^ *p* < 0.05.

^*^ *p* < 0.05, ^*^^*^ *p* < 0.01

When the results of the intergroup comparison of final test scores were examined, the AIDRS score average was very close to the maximum score in the experimental group, while it was close to the average level in the control group, and these differences were statistically significant (*p* < 0.01). The MS score average in the experimental group increased by approximately 14 points due to the effect of ACT and was statistically significantly higher than that of the control group (0.011). The mean scores for the total and subdimensions of the final AMS test in the experimental group were nearly twice those of the control group, and these differences were statistically significant (*p* = 0.011 for AMS total, *p* = 0.003 for intrinsic motivation, and *p* = 0.001 for extrinsic motivation) (Table 3).

**FIGURE 2:**
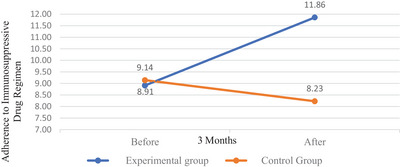
Changes in adherence to immunosuppressive drug regimen of liver transplant recipients in control and experimental groups.

Figure 2 shows the change in compliance with immunosuppressive drug regimens between the experimental and control groups. According to this, ACT administered to the experimental group increased immunosuppressive drug compliance in liver transplant recipients. In the control group, which received no intervention, immunosuppressive drug compliance decreased.

**FIGURE 3:**
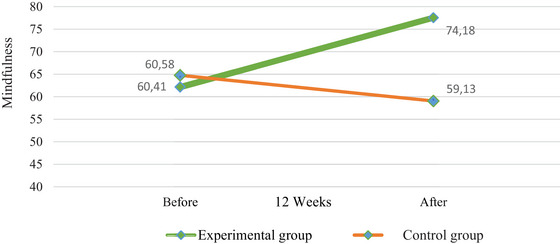
Changes in mindfulness level of liver transplant recipients in experimental and control groups.

Figure 3 shows the change in mindfulness between the experimental and control groups. According to this, ACT applied to the experimental group increased the mindfulness level of liver transplant recipients. The mindfulness level of the control group, which received no intervention, decreased.

**FIGURE 4:**
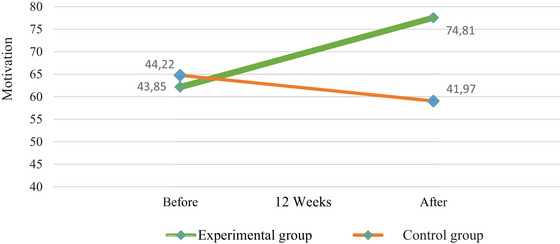
Changes in motivation level of liver transplant recipients in experimental and control groups.

Figure 4 shows the change in motivation levels between the experimental and control groups. In the experimental group where ACT was performed, the motivation levels of liver transplant recipients increased by 1.7 times. The final test motivation score average of the control group, where no intervention was performed, was lower than the pre‐test score.

## 4 Discussion

Transplant surgery appears to be an effective treatment method for eliminating the problems and symptoms caused by chronic liver disease. However, after liver transplantation, recipients begin a new process that must be managed. There is a strong relationship between the effectiveness of the care and treatment required for this process and graft survival. Adherence to immunosuppressive drug regimens represents an important phase in the post‐liver transplant period. The motivation level of liver transplant recipients affects their adherence to immunosuppressive drug regimens. This study demonstrated that the motivation level of post‐liver transplant recipients can be increased, thereby improving their adherence to immunosuppressive drug regimens. In this regard, the main strategy for increasing motivation and medication adherence was ACT. Thanks to ACT, the mindfulness levels of liver transplant recipients increased by almost 25%, while in the control group, where no intervention was made, mindfulness decreased over time. In previous studies, ACT has been used to increase adherence to treatment and disease management. In Pardede's (2019) study, it was found that ACT applied to patients with schizophrenia led to an increase in the ability to accept and adhere to treatment (Pardede 2019). In Rahnama and colleagues' study (Rahnama et al. 2017), ACT was applied to increase medication adherence in coronary heart disease patients. The same study reported reduced psychological distress (depression, anxiety, and stress) and increased medication adherence (Rahnama et al. 2017). As'hab and colleagues' study examined the effect of ACT on the psychosocial adjustment of multidrug‐resistant tuberculosis patients. The same study reported that ACT was effective in reducing anxiety, depression, and suicidal thoughts and increasing treatment adherence compared to standard nursing practice (As'hab et al. 2022). In our study, it is estimated that adherence to an increased immunosuppressive drug regimen in liver transplant recipients is directly related to an increase in mindfulness and motivation levels. In our study, results similar to previous literature were obtained.

Previously, the literature has mentioned that ACT provides high motivation to increase physical activity. Lev Arey and colleagues applied ACT to increase the willingness and motivation of healthy individuals who did not engage in sufficient physical activity to engage in physical activity. The study sample consisted of 94 Israeli university students with low physical activity levels. The same study reported an increase in exercise motivation and intensity at the end of a 12‐week intervention incorporating Self‐Determination Theory and ACT (Lev Arey et al. 2022). A previous study reported that even if individuals intended to engage in physical activity, they were unable to focus on carrying it out and did not do so due to low motivation (Rhodes and de Bruijn 2013).

Through ACT, individuals activate their internal motivation and challenge inconsistent thoughts. A previous study reported that ACT is the most effective mindfulness‐based intervention for promoting health behavior change (C. Q. Zhang et al. 2018). It is clear that ACT develops awareness in liver transplant recipients regarding the necessity and importance of immunosuppressive drug regimens, activates their internal motivation by eliminating inconsistent thoughts, and increases their willingness to cooperate with physicians and nurses, thereby potentially raising their external motivation levels. Indeed, in this study, the motivation level of the experimental group after ACT was nearly 1.7 times that of the control group. Thus, increased mindfulness levels resulted in liver transplant recipients accepting their health status and adhering to the immunosuppressive drug regimen. In the previous two studies, ACT was used to acquire healthy eating habits and smoking cessation behavior (Forman and Butryn 2015; McCallion and Zvolensky 2015). ACT is defined as the ability to engage in value‐based action in the face of incompatible thoughts and feelings, and it is based on the principles of acceptance of the situation and commitment to coping strategies to provide psychological flexibility (Howell and Passmore 2019). ACT supports motivation development by addressing maladaptive thoughts. It facilitates the identification of values and supports individuals in establishing and maintaining consistent connections with their values, thereby enhancing their level of adaptation. The results of our study fully support previous research.

Liver transplant recipients must take immunosuppressive drugs regularly throughout their lives to avoid graft rejection. Regular medication use is of great importance in managing chronic conditions. Patients who do not take their medications regularly are forced to cope with the increasingly severe symptoms of their existing diseases. In a study conducted by Sadeghi et al. (2024) involving dialysis patients, it was reported that patients who did not take their medications regularly had a high clinical symptom burden. In the same study, ACT was tested as an intervention to improve medication adherence. The results of the study showed statistically significant differences between the experimental and control groups in terms of average clinical symptom scores and treatment adherence variables. Additionally, it was found that ACT reduced the clinical symptom burden by improving treatment adherence (Sadeghi et al. 2024). The effect of ACT on medication adherence was examined in a wide range of samples, including patients with diabetes mellitus (Fanayi et al. 2022), mixed anxiety disorder (Arch et al. 2012), and irritable bowel syndrome (Shahkaram et al. 2024), and it was reported that ACT is a highly successful therapy in increasing treatment adherence. Indeed, in this study, the regular medication use achieved by liver transplant recipients through ACT protects them from many potential problems and complications that could arise if they were not taking their medications.

Bulbuloglu and Gunes conducted a study aimed at increasing immunosuppressive treatment compliance after liver transplantation using mindfulness‐based cognitive therapy. In the aforementioned study, the period after liver transplantation was approximately 3 months, and mindfulness‐based cognitive therapy was applied to the experimental group for 8 weeks. It was found that medication compliance had increased in the experimental group 1 month after the intervention. In contrast, liver transplant recipients in the control group, who did not undergo any intervention, showed a further deterioration in medication compliance by the ninth month after transplantation compared to measurements taken 3 months earlier (Bulbuloglu and Gunes 2024). Similar to the findings of Bulbuloglu and Gunes, in this study, the control group of liver transplant recipients showed a statistically significant decrease in adherence to the immunosuppressive medication regimen at the end of 12 weeks compared to the previous measurement. In a study by Zarezadeh et al. (2021), ACT was found to be effective in reducing depression and increasing life expectancy after liver transplantation (Zarezadeh et al. 2021). ACT may be associated with improved psychological well‐being and increased medication adherence. Studies testing ACT in liver transplant recipients are quite limited in number. However, several studies have previously reported that immunosuppressive treatment is not fully adhered to after liver transplantation (Bulbuloglu et al. 2023; Cinar and Bulbuloglu 2022; Demir and Bulbuloglu, 2021; Gunes et al. 2022), and these same studies shed light on the need for this study to develop strategies to improve adherence to immunosuppressive treatment. Prior to this study, no application had been made to screen the psychological resilience of liver transplant recipients. The data collection tools used were self‐report‐based, and no objective tests were conducted to determine whether immunosuppressive drugs were metabolized in the body. The small sample size and the fact that this was a single‐center study limit the generalizability of the results to the population. The outcomes of liver transplant recipients in the experimental and control groups after the final test measurement were not investigated. Family, occupational, and social problems that may reduce medication adherence in liver transplant recipients were not examined. All of these were accepted as limitations.

## 5 Conclusion

After liver transplantation, recipients enter a process that requires them to prioritize their health at all times. During this process, liver transplant recipients must make a significant effort to ensure that graft function improves and increases day by day. To this end, it is of great importance that they are motivated to adhere closely to their immunosuppressive drug regimen. Increasing the motivation of liver transplant recipients and improving their adherence to immunosuppressive drug regimens highlights the need for strategic approaches to maintaining graft function and survival. In this study, acceptance and commitment therapy applied to liver transplant recipients improved adherence to immunosuppressive drug regimens by increasing mindfulness and motivation. As adherence to immunosuppressive drug regimens decreases in liver transplant recipients, the risk of complications and death increases. The addition of acceptance and commitment therapy to the multi‐care practices taught to liver transplant recipients provides significant advantages in terms of being a non‐pharmacological intervention and supporting recipients in coping with their conditions. We recommend that liver transplant recipients undergo acceptance and commitment therapy to improve their adherence to immunosuppressive medication and increase their motivation to do so.

## Author Contributions

**Serdar Saritas**: writing – original draft, funding acquisition, data curation, conceptualization, methodology, investigation. **Serafettin Okutan**: project administration, methodology, conceptualization, formal analysis, data curation, project administration, resources. **Hasan Saritas**: project administration, software, conceptualization. **Semra Bulbuloglu**: writing – review and editing, conceptualization, methodology, validation, supervision, visualization.

## Ethics Statement

Prior to the start of this study, Institutional Review Board approval was obtained from the Turgut Özal Medical Center Liver Transplant Institute. Subsequently, Ethical Committee approval was obtained from the Bitlis Eren University Ethics Committee (Date: February 13, 2025, Decision No: 2025/1‐2). In the research protocol, informed written consent was obtained from each liver transplant recipient, taking into account compliance with the Helsinki Declaration. Additionally, the study protocol was recorded on the Clinical Trials Platform with the registration number NCT06879106.

## Conflicts of Interest

The authors declare no conflicts of interest.

## Peer Review

The peer review history for this article is available at https://publons.com/publon/10.1002/brb3.70882

## Data Availability

The data that support the findings of this study are available on request from the corresponding author. The data are not publicly available due to privacy or ethical restrictions.
